# Experimental and simulation study on compressive failure of rock with pre-Y-shaped cracks

**DOI:** 10.1371/journal.pone.0312344

**Published:** 2024-11-14

**Authors:** Chao Peng, Xiaotong Du, Zhan Li, Wanrong Liu, Bin Huang

**Affiliations:** College of Architecture and Engineering, Liaocheng University, Liaocheng, Shandong Province, China; Shenyang Jianzhu University, CHINA

## Abstract

A large number of joints and fissures are prevalent in the rock mass, which has an important influence on the mechanical properties of the rock mass. To study the failure mechanical characteristics of Y-cracked rocks, the paper analyzes the influence of different angles of prefabricated Y-cracked rocks on the mechanical strength characteristics of the rocks and the crack extension evolution through uniaxial compression indoor tests and discrete element PFC^2D^ numerical simulation. The results indicate that the stress-strain curves of rocks containing prefabricated cracks exhibit five stages: the initial pore-fracture compaction stage, the elastic stage, the crack stable development stage, the crack unstable development stage, and the post-peak rupture. The peak strength of the specimen shows an evolutionary process of decreasing, then increasing, and then decreasing with the increase of the Y-shaped crack angle. The failure of the sample is mainly caused by the shear crack expansion at the crack tip. The different Angle cracks directly affect the mechanical properties of the sample and the generation and evolution of new cracks. The final failure of rock is mainly the result of microcrack propagation, convergence and penetration to form macroscopic damage zone. Finally, combined with PFC numerical simulation, the distribution of micro-cracks and the damage pattern of rock damage are compared and analyzed, and it is found that the two are in good agreement, which reflects the rationality of the model.

## Introduction

With the rapid development of China’s economic modernization level and the need for population growth, the exploration and comprehensive utilization of underground space resources and the construction of underground engineering are of great significance and value to the development and construction of China. In the process of deep underground engineering, activities are mainly aimed at rock materials. Therefore, it is of great significance to carry out experimental research on the strength and deformation damage of defective rocks and to understand the damage and destruction characteristics of rock materials to guide engineering practice.

Rock damage analysis is a classical topic in the field of rock mechanics research. There are two main methods to study the mechanical properties of rocks containing defects, namely, indoor testing and numerical simulation. In indoor testing, the most common method for studying defective rocks is through a combination of high-speed photography, CT scanning, and digital imaging methods. Numerical simulation, on the other hand, has been widely used in many fields of engineering and science, and great progress has been made in numerical simulation of nonlinear and discontinuous problems in geomechanics and rock mechanics. Meanwhile, acoustic emission is an effective means to monitor the real-time breakage process inside rock materials and has been widely used in the analysis of the crack extension process inside many intact rock materials [1, 2].

Liu et al. [3] investigated the effects of different clay layer thicknesses, layer positions, quantities and moisture contents on the mechanical properties of mudstone-clay composites by uniaxial loading tests; Xiao et al. [4] in order to investigate the effects of internal defects in intact granite on the prestressing force, the mechanical response and the damage behavior of the rock under impact loading, the improved Separate Hopkinson Pressure Bar (SHPB) and Digital Image Correlation (DIC) methods for specimens and specimens with different defect inclinations; Yang et al. [5] developed a reasonable numerical model for non-persistent joint-anchored rock samples based on indoor rock samples using a three-dimensional particle flow program (PFC) and conducted numerical uniaxial compression tests; Yang et al. [6] investigated a granite specimen containing two non-coplanar open fissures using a conventional triaxial compression test. In terms of numerical simulation, Abaqus, PFC, RFPA, and other software were used for the study, and the global strain field evolution law of cracks was experimentally obtained and found that the rock containing inclined cracks under uniaxial compression would undergo wing crack expansion and gradual damage destruction. Wang et al. [7] used PFC2D to analyze the numerical model, established 7 combined models of single circular hole and double crack with different angles, conducted uniaxial compression tests, and studied the crack development law and acoustic emission characteristics of different defect combined models. Wang et al. [8] used laboratory test data to calibrate the micromechanical parameters required for particle flow program (PFC) simulation, and studied the evolution process of cracks and stress field in round, square, triangular and trapezoidal cavity coal under uniaxial compression in order to study the influence of different shapes of cavity defects in coal rock on its mechanical behavior and macroscopic damage law. Wang et al. [9] designed and carried out CO2 fracturing experiments on the new sealing test platform, determined the influence radius of the crushing zone, fracture zone and elastic zone, and studied the change of energy distribution. Li et al. [10] developed a two-dimensional rock constitutive model including strain-related elastic modulus, which can effectively represent the nonlinear stress-strain curve using only three equations and four parameters, and implement the simple two-dimensional model as finite element code to solve engineering problems. Many scholars, such as Zhu [11] and Shi [12], have also studied rocks containing various types of intersecting type joint fractures.

**Fig 1:**
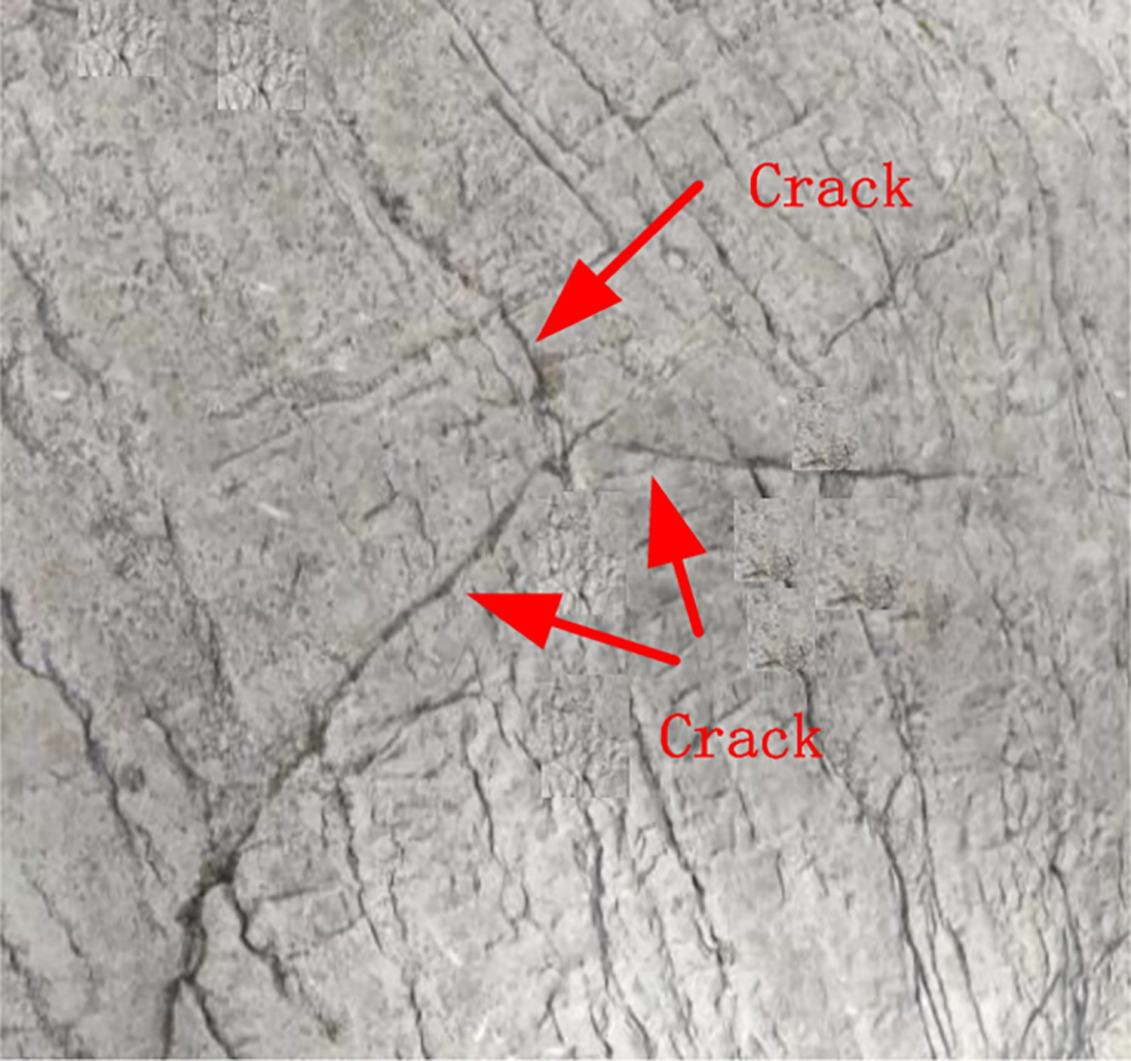
Physical diagram of Y-shaped crack.

In fact, for brittle materials such as rock mass, when there are tiny pores in the rock mass, the cracks will expand along the pores in all directions, forming an emission-like Y-shaped fracture, as shown in Fig 1. As a special microstructure in rocks, Y-shaped defects are closely related to complex stress processes, fluid movements or mineral compositions. As a weak link in rock failure, Y-shaped defects often become a stress concentration area, which affects the overall strength and stability of rock. In geological engineering, coal mine exploration and other fields, understanding the distribution and characteristics of Y-shaped defects in rocks is helpful to evaluate the safety and stability of the project, and can reveal the complex stress processes occurring in the interior of rocks. However, the current research on the fracture mode and damage mechanism of the rock body containing nodal fissures is mostly based on a single fracture or multiple fractures distributed in parallel, and there are fewer studies on the macro fine-scale evolution process of crack extension, rock damage mode, and mechanical characteristics of the rocks containing prefabricated Y-shaped cracks under compression conditions. Therefore, this paper adopts a combination of indoor experiments and PFC numerical simulation to study the Y-shaped crack rocks, to reveal the influence of Y- shaped cracks on rock damage and the evolution of crack extension.

## Indoor test and equipment

### Specimen preparation and test program

Natural rock mass contains irregular joint cracks. In response to the distribution of Y-shaped cracks in natural rock mass, research on the mechanical properties of Y-shaped crack rock mass is carried out. In this study, high-pressure hydraulic cutting technology is used to form Y-shaped cracks on rock specimens. In actual operation, special high-pressure hydraulic cutting equipment is used, which includes high-pressure pumps, nozzles, control systems and other key components. By precisely controlling the output pressure of the high pressure pump and the moving track of the nozzle, the Y-shaped crack can be precisely cut. At the same time, in order to further improve the cutting accuracy and efficiency, computer aided design and manufacturing (CAD/CAM) technology is used to optimize the cutting path and parameters. During the cutting process, a number of preventive measures are taken to prevent microscopic damage to the sample, such as selecting the appropriate nozzle material and shape, and strictly controlling the cutting parameters. The study begins with a uniaxial compression test conducted on Y-shaped cracks rock specimens to understand their fracture initiation pattern and mechanical characteristics. To ensure the accuracy of the test results, several steps are taken. Firstly, when selecting rock specimens, efforts are made to avoid rocks with more defective structures to reduce data dispersion. Secondly, sampling and processing are carried out on the same rock mass to maintain consistency. Lastly, the end faces of the specimens are carefully polished to ensure flatness, which is crucial for accurate test results.

**Fig 2:**
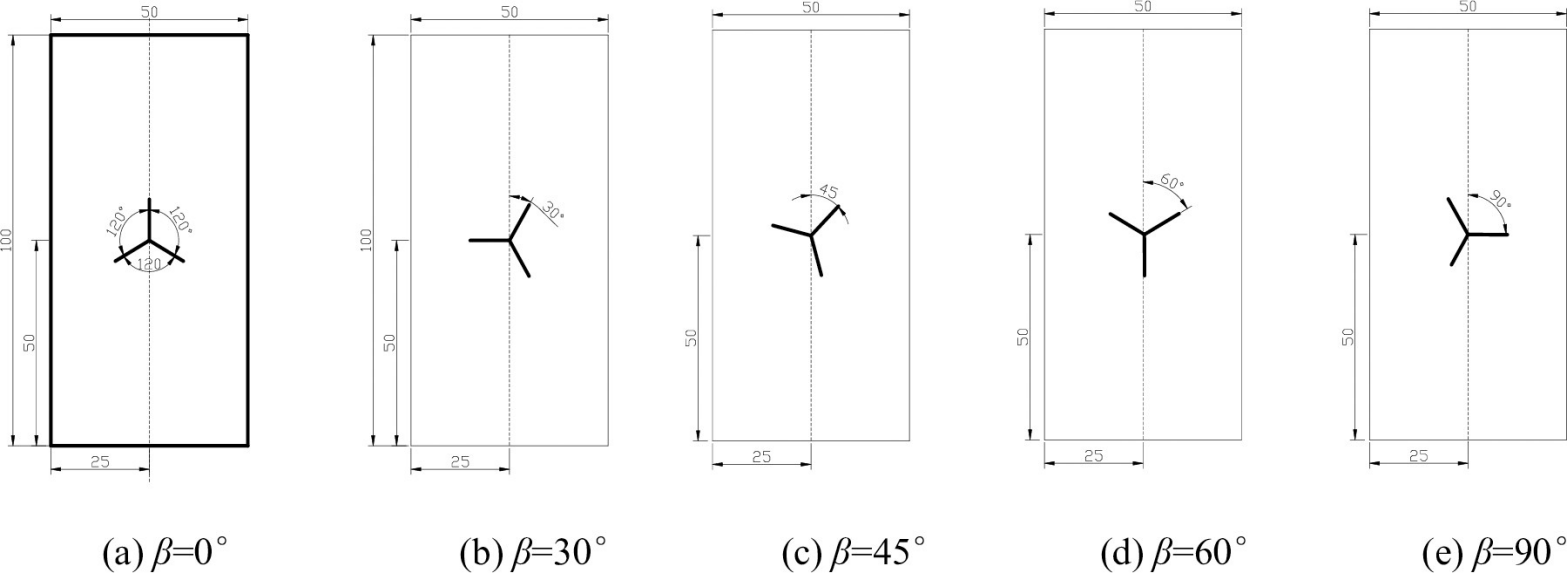
Schematic diagram of Y-shaped crack rock specimen.

**Fig 3:**
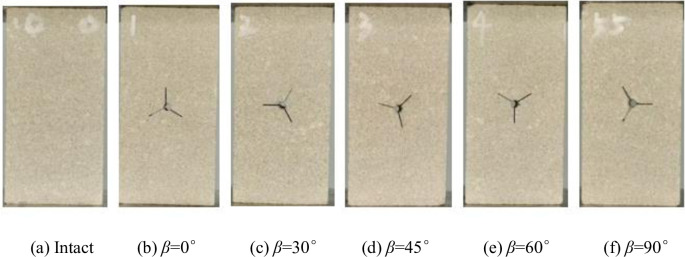
Physical drawing of Y-shaped crack rock specimen.

The specimens were made of homogeneous fine sandstone, and the same rock was used to sample the same group of specimens, the length and height of the specimens and the thickness of the specimens were 50mm * 100mm * 25mm. The rock is limestone with a density of 2.48g/cm^3^. Three cracks were cut in the center position, each crack was 120°from each other, the length of each crack was 10mm, and the width of each crack was 1mm. Point O was the center of the cracked joints of the specimens, and the three bifurcated cracks of the Y-shaped cracks were named a, b, and c respectively, and the angle of crack a to the vertical direction was β. The angle between crack *a* and the vertical direction of the specimen is β. To realize different working conditions, β is 0°, 30°, 45°, 60°and 90°, respectively. The final five experimental group specimens plus one control group of crack-free intact specimens totaled six, as shown in Figs 2 and 3.

### Test equipment and process

**Fig 4:**
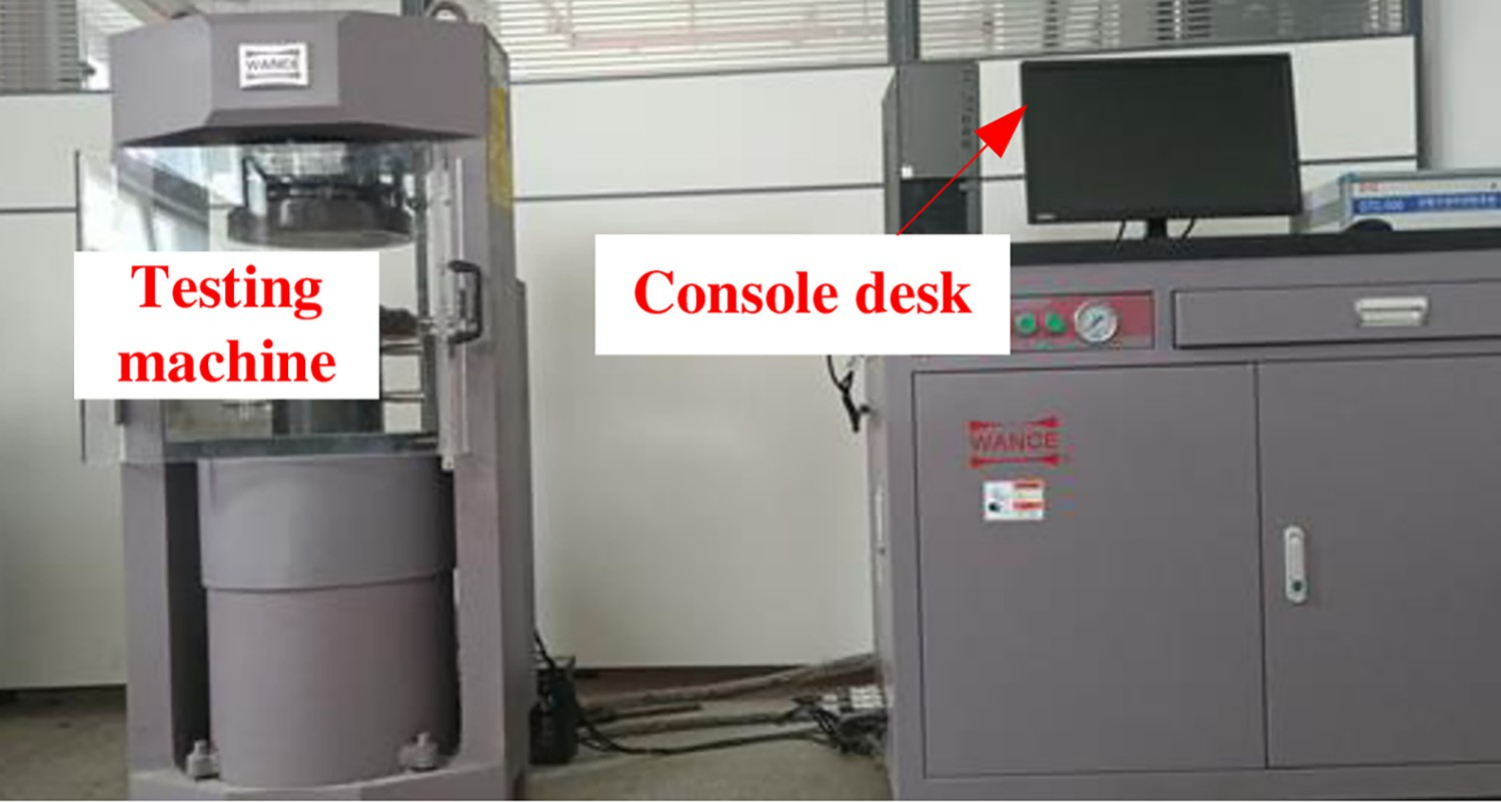
Test equipment.

The electro-hydraulic servo rigid rock mechanics testing machine from Liaocheng University’s Civil Engineering Experimental Center, as shown in Fig 4 [13], showcases several noteworthy features. These include substantial axial stiffness, precise testing accuracy, stable performance, and reliability. The machine facilitates control through both force and displacement loading mechanisms. In the present test, displacement loading was employed at a rate of 0.05 mm/min. To initiate the test, a manual triggering system was activated to ensure accurate time recording. Recording ceased upon the occurrence of specimen damage, at which point the gathered data was stored for further analysis. This meticulous approach ensures the integrity of the test results and allows for comprehensive evaluation and interpretation.

## Analysis of test results

### Mechanical characteristics of Y-shaped crack rock failure

**Fig 5:**
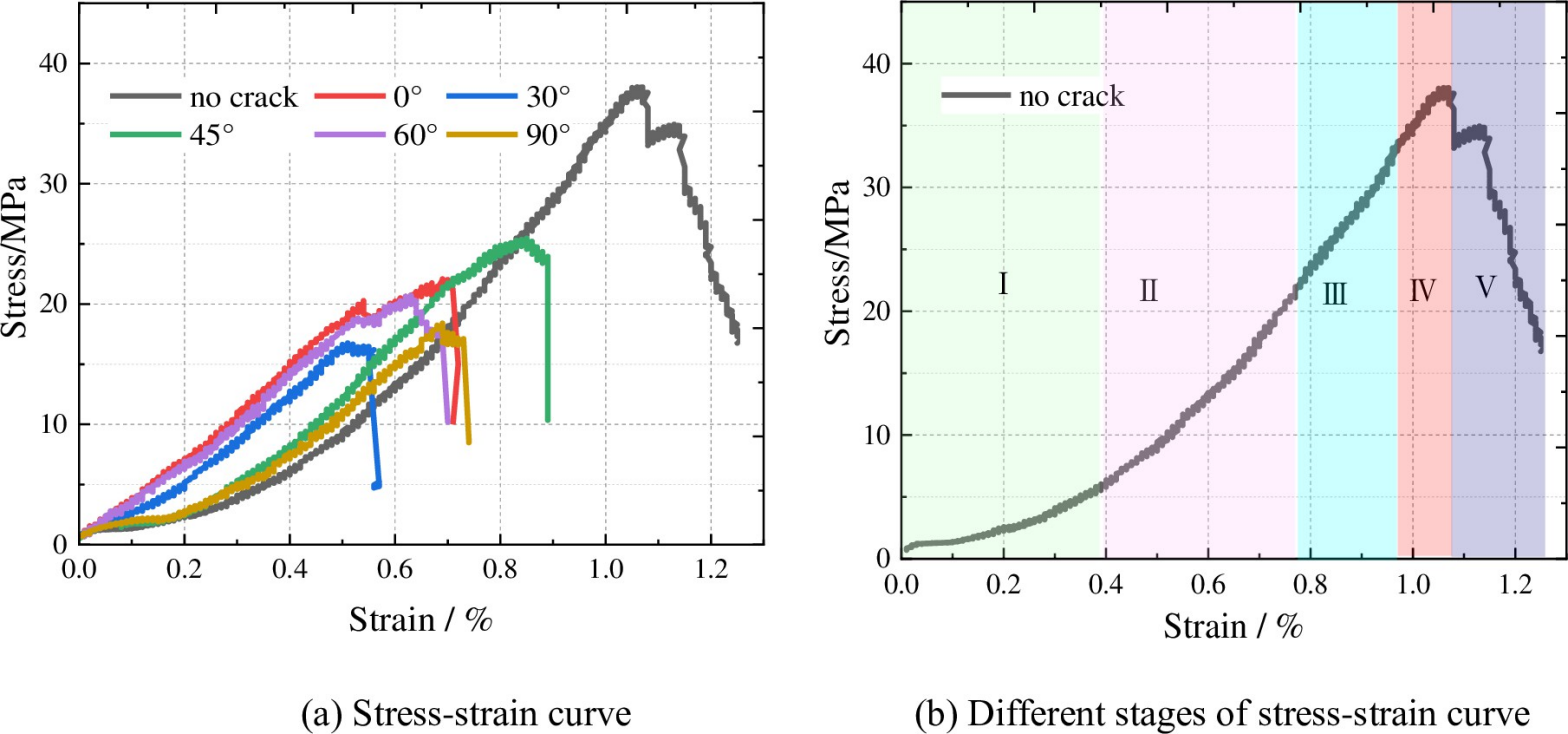
Stress-strain curve and different stages of curve.

The stress-strain curves of Y-shaped crack rocks were obtained by uniaxial compression failure test as shown in Fig 5. From Fig 5, it can be seen that the stress-strain curves of Y-shaped crack rocks exhibit five distinct stages. Initial pore-fracture compaction stage, with the increase of axial load, the specimen volume decreases with the increase of load, and each group of specimens in the rock specimen stress-strain curve due to the initial existence of internal pore fissures is compressed and compacted in an upward concave type, the specimen radial expansion of the smaller. In the elastic stage, with the increase of axial load, the stress-strain curve is approximately straight. In the stable crack development stage, the slope of the stress-strain curve decreases with the increase of stress, and new fissure cracks begin to be produced inside the specimen at this stage, but the development is stable due to the control of the applied load. In the non-stationary crack development stage, the stress-strain curve shows an upward convex trend, and the cracks continue to develop in this stage until the specimen is completely destroyed and the stress reaches the peak intensity. In the post-peak rupture stage, the bearing capacity of the rock decreases rapidly with the increase of strain, but there is some residual strength at the end. It can be seen that the destruction of the specimen is mainly the result of the generation of new cracks, the steady development of cracks, the rapid development of cracks, and the formation of a macroscopic damage surface by the convergence and penetration of cracks, which ultimately leads to the loss of the bearing capacity of the specimen.

**Fig 6:**
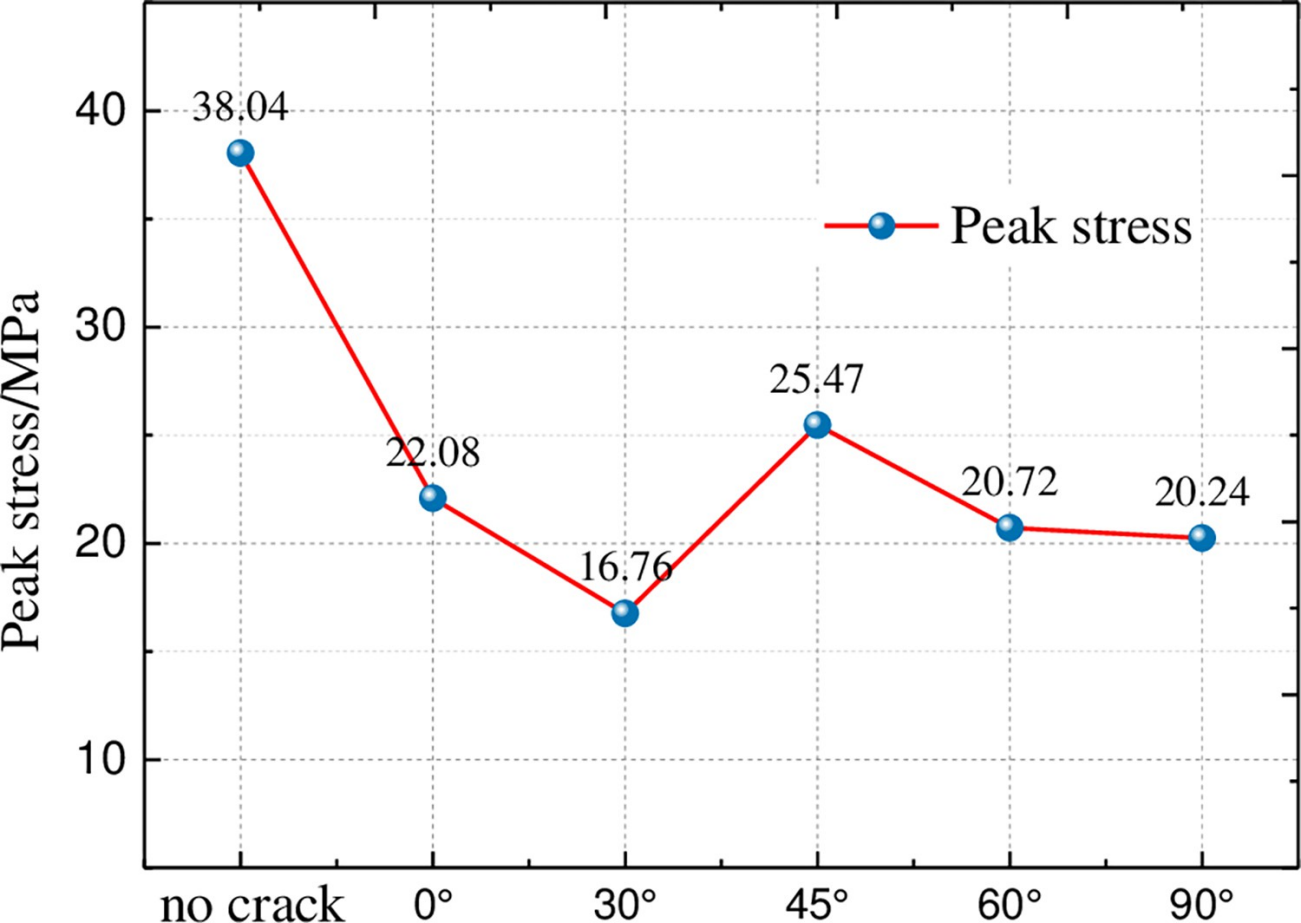
Peak strength curve.

Fig 6 shows the peak strength curves of each group of specimens, according to the figure can be obtained: the peak intensity with the increase of the crack offset vertical direction angle in the Y-shaped crack appeared to be firstly reduced and then increased, and then reduced the evolution process. The peak intensity of the intact specimen is 38.04MPa, and the peak intensity is 22.08MΠα when the angle of the Y-shaped joint is 0°, and the peak intensity decreases by 41.9%; the peak intensity is 16.76MΠα when the angle is 30°, and the peak intensity decreases by 55.8%; the peak intensity is 25.47MΠα when the angle is 45°, and the peak intensity decreases by 32.9%; the peak intensity is 20.72MΠα when the angle is 60°, and the peak intensity decreases by 45.4%; when the angle is 90° the peak intensity is 18.24MΠα, and the peak intensity decreases by 52.1%. The angle of 30° and 45° is the turning point of the peak strength of the specimen, when the offset angle is less than 30° and more than 45°, the peak strength of the specimen decreases with the increase of the angle, and the angle is 30°, the specimen strength is the lowest. It can be seen that the peak strength is related to the fissure defects of the specimen, and the different forms of Y-shaped crack distribution directly affect the mechanical characteristics of the specimen.

### Analysis of Y-shaped crack rock failure mode and macroscopic crack extension

**Fig 7:**
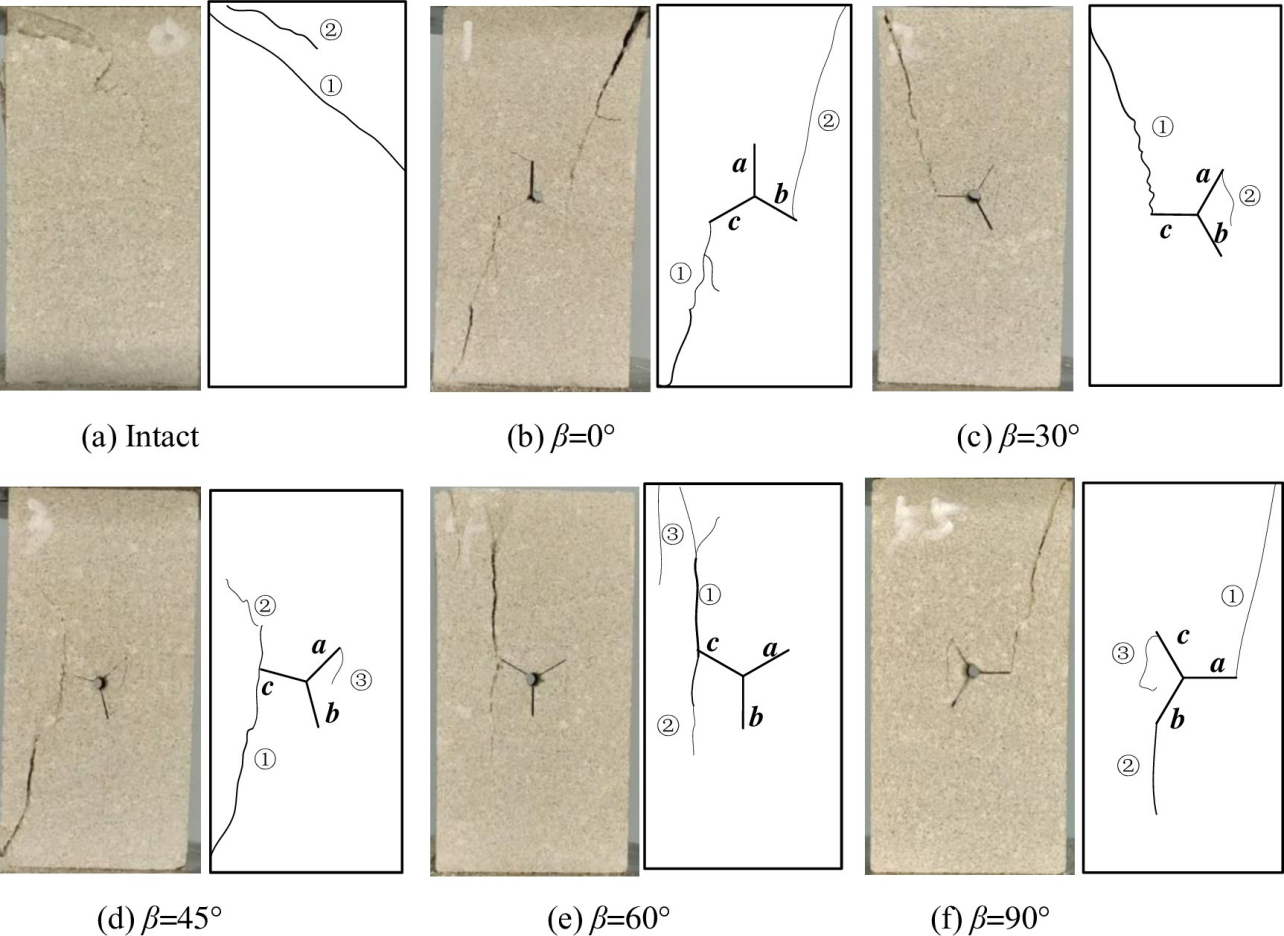
Rock failure mode.

The failure mode and crack sketch of the specimen are shown in Fig 7, and the numbers in the figure are the crack numbers. When the rock is intact, the failure to the rock is mainly dominated by crack No. ①, which extends from the upper left corner to the middle of the right side to form a penetrating inclined shear damage zone, and the specimen loses its load-bearing capacity. In the main crack expansion process to form ② crack. When the vertical deviation angle of pre-crack a in the Y-shaped crack of the specimen is 0, shear cracks ① and ② are formed at the tips of pre-crack b and c. Crack ① extends to the lower left corner along pre-crack c, and crack ② extends to the upper right corner along pre-crack b. Cracks ① and ② penetrate, causing the entire specimen to lose its load-bearing capacity. When the pre-crack a in the Y-shaped crack of the specimen is offset from the vertical direction at an angle of 30, the shear crack ① is formed along the pre-crack c and the tensile crack ② is formed along the pre-crack a, and the specimen is mainly damaged due to the expansion of crack ①. When pre-crack a in the Y-shaped crack of the specimen deviates from the vertical direction by an angle of 45, shear cracks ① and ② are formed along pre-crack c, and crack ③ is formed along pre-crack a. The crack propagates along the axial loading direction, and the failure of the specimen is the result of shear crack penetration failure. When the specimen Y-shaped crack in the crack an offset vertical direction angle of 60, in the pre-crack c upward to form tension crack ①, while expanding downward to form tension crack ②, in the upper left side of the specimen to form a localized tension crack ③, cracks are also along the axial loading direction of the expansion of cracks through the specimen ultimately lead to specimen damage. When the pre-crack a in the Y-shaped crack of the specimen is offset from the vertical direction at an angle of 90, the tension crack ① is generated along the crack a to the upper right, the tension crack ② is generated along the pre-crack b downward, and the crack ③ is generated along the pre-crack c. The damage to the specimen is mainly caused by the tension crack in the lower left corner and the shear crack in the upper right corner which extends and penetrates through the specimen.

The crack of specimen starts to crack at the tip of the prefabricated Y-shaped crack, and the crack expands outward from the tip, and the new crack produced is mainly shear, and with the change of the angle of the Y-shaped crack, the location of crack initiation and the expansion of the crack evolution also change with it. It can be seen that the destructive failure of the specimen is mainly caused by the shear crack, and the strength of the specimen is related to the angle of the crack offset from the vertical direction in the prefabricated Y-shaped crack.

## Numerical simulation study of Y-shaped crack rock failure

Sandstone is a sedimentary rock formed by clastic particles through cemented material, and the particles and cemented material inside the sandstone can be seen at a certain magnification. From the figure, it can be seen that the debris particles play the role of skeleton support, and cemented material to maintain the stability of the skeleton, the two together determine the bearing capacity of the rock material. A large number of studies have shown that the destruction of sandstone is mainly due to the destruction of the cementation between the particles leading to the failure of the rock. Particle flow code (PFC) in the early application is mainly used to simulate the bulk particles, with the continuous deepening of the research, scholars found that the particles through the cementation effect can realize the simulation of solid material analysis, especially in the fine mechanical analysis has certain advantages. The significant advantage of using PFC to simulate rock uniaxial compression experiment is that it can accurately capture the non-uniformity, dispersion and nonlinear mechanical behavior of rock materials at mesoscale, so as to more truly simulate the whole process of rock failure, including the complex mechanism of crack initiation, expansion and final failure, and provide strong support for in-depth understanding of rock mechanical properties. Since entering the 21st century, the research of particle discrete elements in geotechnics has made a lot of research results and has been widely used [14–20]. Particle discrete elements are formed by bonding particulate matter to form aggregates through the parallel bonding model. When the aggregate is under external force, the parallel bond is damaged to form microcracks, and with the expansion of the microcracks, macroscopic damage occurs through the cracks. It can be seen that the damage mechanism of the rock material is similar to that of the discrete element parallel bonding model of particles. Therefore, to further study the damaged fine mechanical characteristics of Y-shaped crack rock, the following particle discrete element PFC is used to study it.

### Modeling and parameter calibration

**Fig 8:**
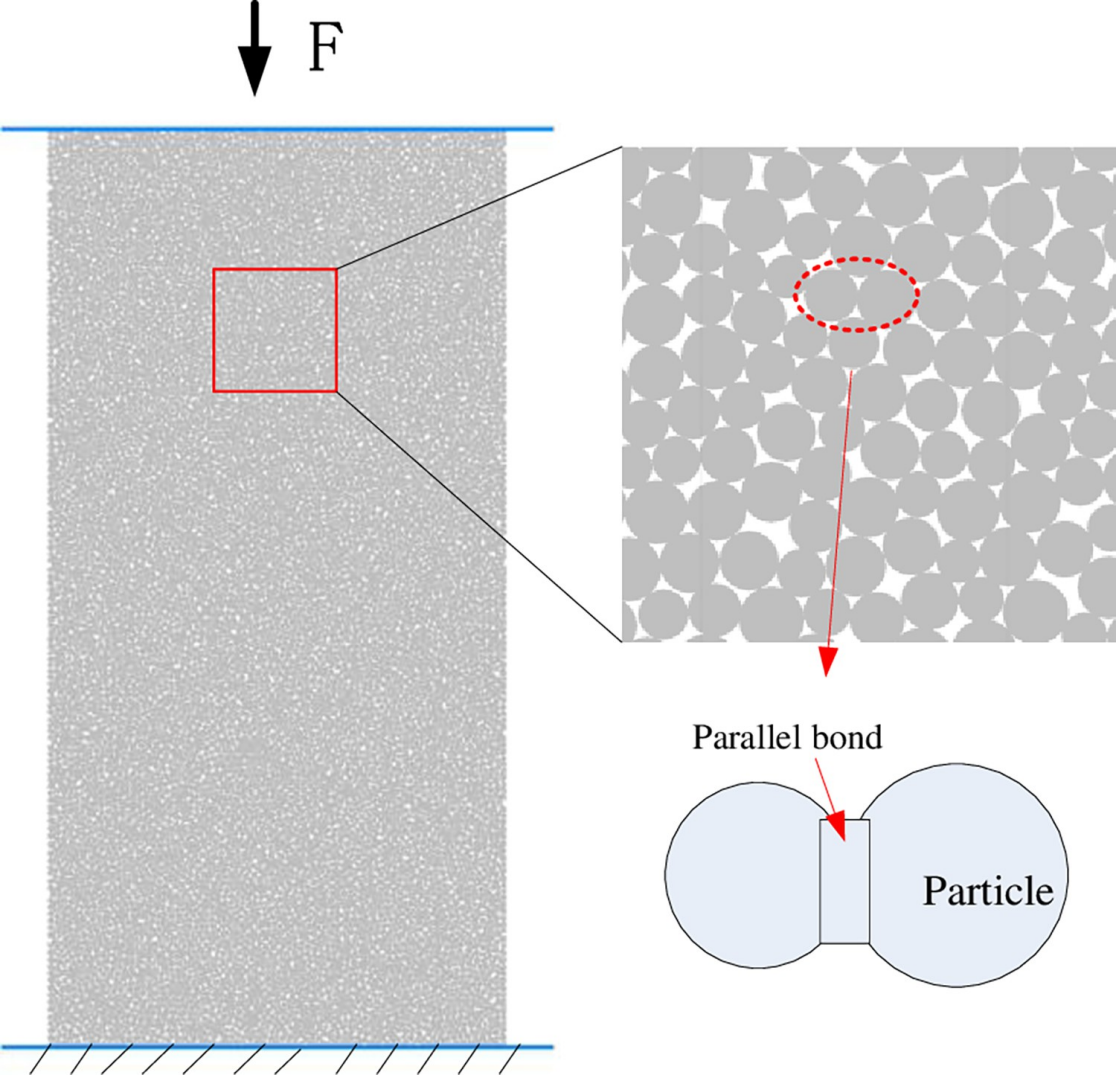
Uniaxial compression model and parallel bond.

The establishment of a realistic numerical analysis model is the basis of the study, to maintain the same size as the actual specimen, the PFC^2D^ software was used to establish a model with a length * width of 100 mm * 50 mm, as shown in Fig 8. First of all, four walls were generated, and then particles of different sizes were generated in the walls according to the normal distribution, with the maximum particle size of 0.45 mm and the minimum particle size of 0.3 mm; then the particles and the bond were parameterized to obtain the numerical analysis model. Through direct tensile and Brazilian splitting tests, Yu et al. [18] show that the parallel bond model is consistent with the tensile and compressive strength ratio of most rock materials. Therefore, in this test, the parallel bonding model can be closer to the actual sample state. The force transfer inside the model is mainly realized by the contact force chain between the particles, and the parallel bond is used for the bonding key in the simulation process, which can transfer both force and moment and is more in line with the mechanical characteristics of rock-like materials. As shown in Fig 8.

**Fig 9:**
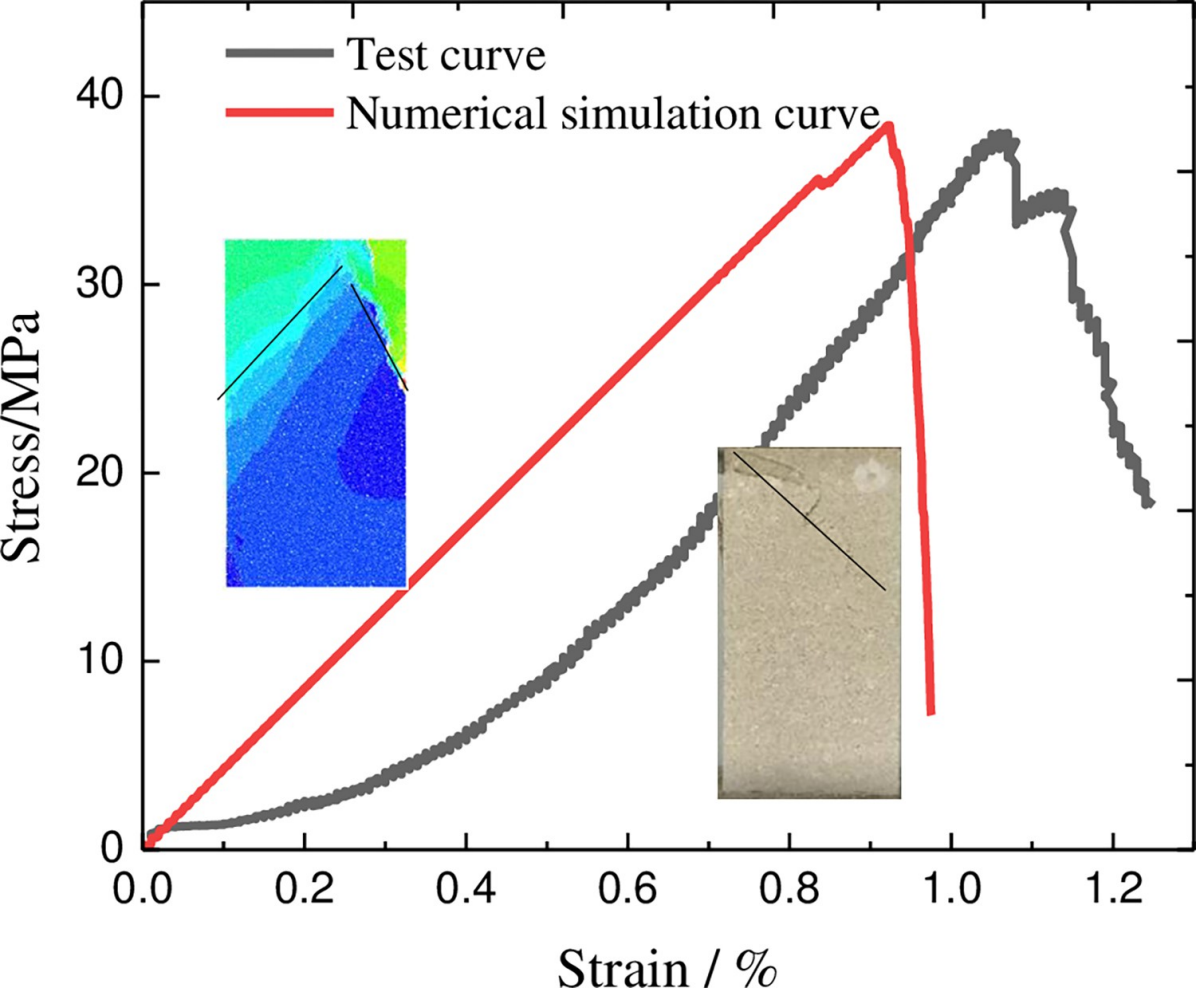
Stress-strain curves for tests and numerical simulations.

The setting of the specific parameters of the model is a difficult point in the analysis of the PFC model, and this paper analyzes the parameters of the previous research methods [17, 18]. At present, the simulated fine-scale mechanical parameters are mainly obtained by matching the uniaxial compression test stress-strain curve in the chamber and the uniaxial compression test stress-strain curve of the numerical analysis model, as shown in Fig 9. In this paper, through a large number of trial calculations and repeated adjustment of particle fine view parameters, the specific fine view mechanical parameters of the sandstone PFC model were finally determined as shown in Table 1. The full stress-strain curve of sandstone simulated by indoor test and numerical calculation shows that the peak stress of the numerical simulation curve is 38.75MPa, and the elastic modulus is 4.26GPa; the peak stress of the test is 38.04MPa, and the elastic modulus is 4.35GPa. It can be seen that the main mechanical parameters of the two are in good agreement, and the microscopic mechanical parameters can better respond to the macroscopic mechanical characteristics.

**Table 1:** Particle mechanical parameters.

Parameter	Value	Parameter	Value
deform emod /MPa	4.2*10^3^	pb_deform emod/MPa	4.2*10^3^
density (kg/m^3^)	2480	pb_fa	38°
krat	1.8	gap	0.5e^-4^
pb_ten /MPa	13.2	pb_coh /MPa	14.2

### Numerical simulation program

**Fig 10:**
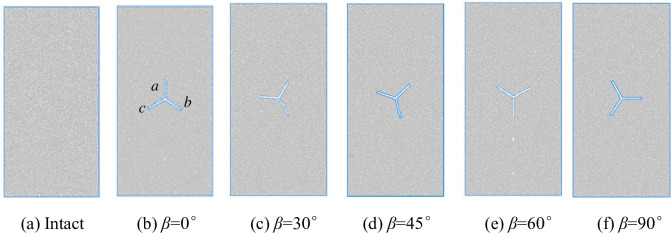
Numerical model of Y-shaped crack rock.

A detailed numerical simulation scheme was developed based on the indoor test scheme, and to further analyze the damage characteristics of the rock containing prefabricated Y-shaped crack from a microscopic point of view, a numerical simulation scheme corresponding to the indoor test was established, as shown in Fig 10. Where *β* is 0°, 30°, 45°, 60° and 90°, respectively, when *β* is 0°, its crack a is coincident with the axis of the specimen, 120° between each crack, the length of each bifurcated crack is 10 mm, and the width of the crack is 1 mm. Crack extension and damage characteristics of prefabricated Y-shaped crack with different angles are analyzed under uniaxial compression.

### Analysis of numerical simulation results

#### Characteristics of specimen damage displacement field distribution

**Fig 11:**
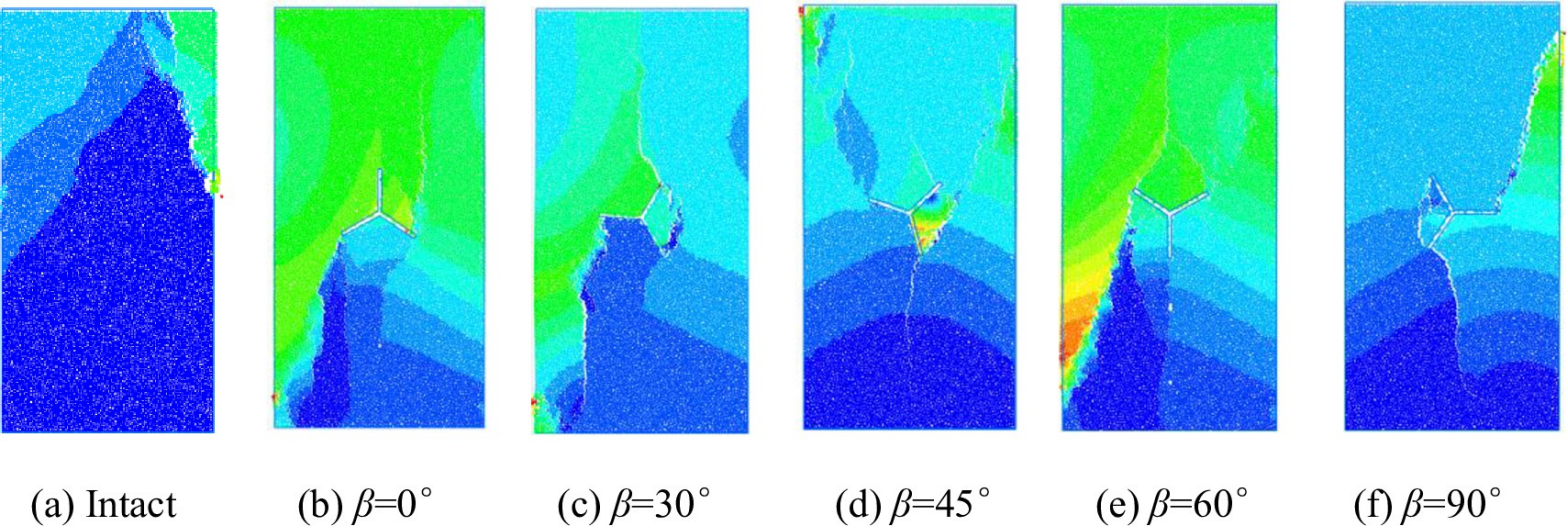
Specimen displacement distribution.

The displacement field of the particles in the PFC model is used to characterize the damage of the specimen, as shown in Fig 11. The color in the figure indicates the size of the particle displacement: the darker the blue indicates the smaller the displacement, the deeper the red indicates the larger the displacement, and if there is a significant color difference between the specimens indicates that the specimen block has a large sliding displacement between the blocks, that is to say, indicates that there is a crack at this location. The color difference in Fig 11(A) is spike-like and mainly distributed in the middle and upper part of the specimen, and the damage of the specimen is mainly caused by the expansion of the cracks in the middle position of the right side to the upper left side. The color difference in Fig 11(B) is mainly distributed in the lower-left part and lower-right part of the Y-shaped crack, and the damage of the specimen is mainly caused by the expansion of the cracks b to the upper right side and the cracks c to the lower left corner. The color difference in Fig 11(C) is mainly distributed near pre-crack a, towards the lower left corner of pre-crack c, and in the area between pre-crack a and b. The failure of the specimen is mainly caused by the tilted cracks running through the specimen; the obvious color difference in Fig 11(D) is mainly distributed in the vicinity of Y-shaped crack, especially in the area between pre-crack a and b, and the area between the pre-crack c to the upper left corner, and the damage is mainly caused by the tilted cracks running through the specimen along the pre-crack b and c. Fig 11(e) is mainly distributed in the lower left area and shows obvious large red areas, indicating that there is a spalling phenomenon. The damage to the specimen is mainly caused by the inclined cracks along the pre-crack b and c. The obvious color difference in Fig 11(E) is mainly distributed in the lower left part of the specimen and shows large red areas, which indicates that there is a spalling phenomenon. The damage of the specimen is mainly caused by the inclined crack in the lower left part; the obvious color difference in Fig 11(F) is mainly distributed in the direction from pre-crack a to the upper right and from pre-crack b to the axis, and the damage of the specimen is mainly caused by the inclined crack running through the specimen. It can be seen that the rupture pattern of the specimen is consistent with the damage pattern of the indoor test.

#### Characteristics of damage microcrack distribution in Y-shaped crack rocks

**Fig 12:**
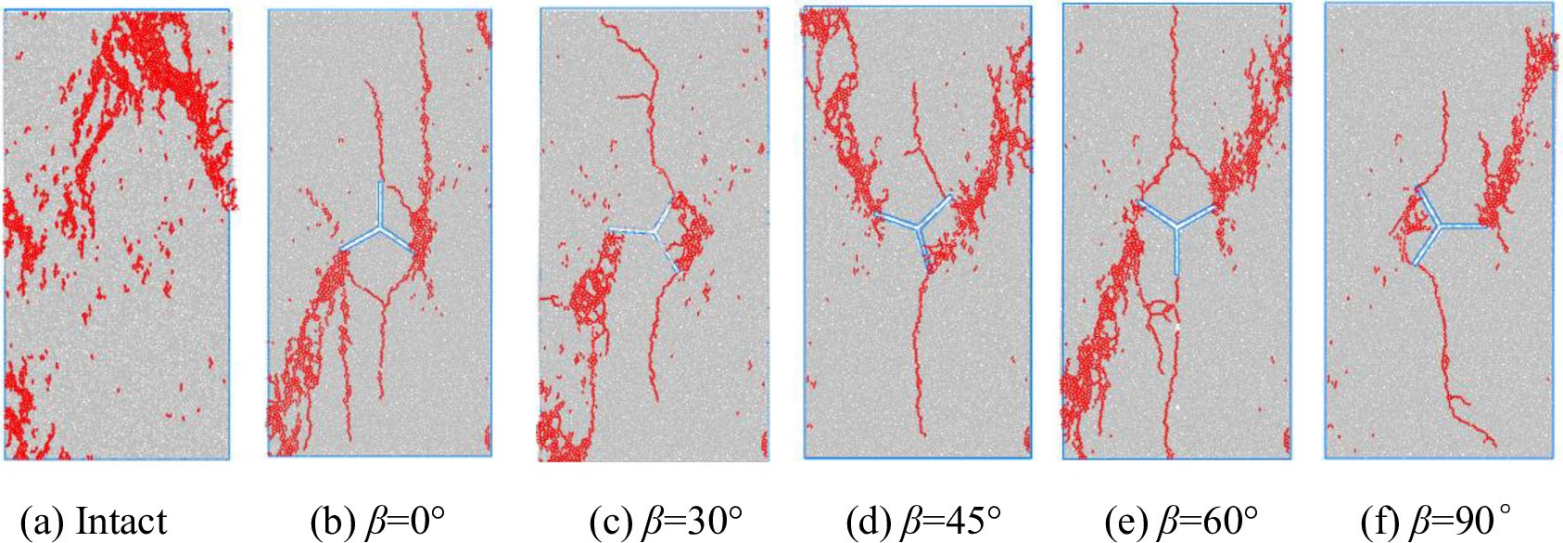
Distribution of microcracks in the specimen.

Fig 12 shows the microcrack distribution pattern of the specimen, and the red color indicates the specimen microcracks. From the figure, it can be concluded that when the applied load is greater than the bond strength between the particles, the bond chain breaks to form microcracks, the local sporadic microcracks have a small effect on the strength of the specimen, and when a large number of microcracks are aggregated, they will be through the rows of the damage zone, which ultimately results in the loss of the load-bearing capacity of the specimen. Fig 12(A) cracks are mainly concentrated in the upper-middle and lower-left parts, which are in the form of spikes, with a small number of localized microcracks. Fig 12(B) cracks are mainly concentrated in the direction of pre-crack b upward toward the axis as well as in the direction of pre-crack c toward the lower-left corner, which forms a damage zone. Fig 12(C) cracks are mainly concentrated between pre-crack a and b and in the lower-left part of pre-crack c, and there are consecutive microcracks expanding along the direction of specimen axes starting from the ends of pre-crack a and b. Fig 12(D) cracks are mainly concentrated in the vicinity of pre-crack a and b and to the upper right, and pre-crack c to the upper left, there are also continuous microcracks along the specimen axis. Fig 12(E) there are microcracks through the direction of the specimen axis, the cracks are mainly concentrated in the pre-crack a to the upper right, and pre-crack c to the lower left to form a damage zone. Fig 12(F) cracks are mainly concentrated in the pre-crack a to the upper right to form a damage zone, and the area between the nodules b and c is also more intensive. The microcracks in the region between the pre-crack are also denser. It can be seen that the damage of the specimen is a result of the development of microcrack damage to a certain extent and then the formation of macroscopic damage zones, leading to the final formation of the specimen shear or tension damage zone.

#### Contact force chain distribution

**Fig 13:**
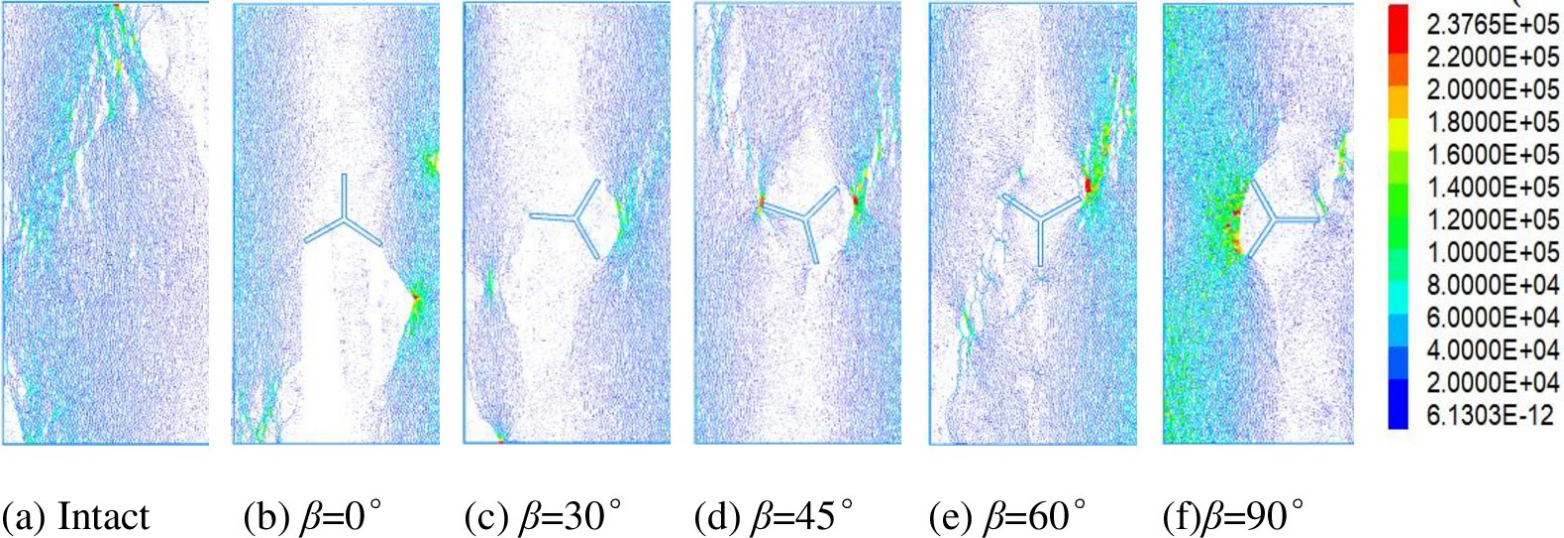
Distribution of contact force chain of specimen.

The applied load is transferred in the specimen through the contact force chain between the particles, and analyzing the distribution of the contact force chain can be used to comparatively analyze the effect of Y-shaped crack on the uniaxial compression test of rocks from a fine-grained point of view. The force chain distribution of rock specimens with Y-shaped crack at different angles is shown in Fig 13, where the colors indicate the magnitude of the contact force: the deeper the red color indicates the larger the contact force and the more concentrated the force, and the deeper the blue color indicates the smaller the contact force. From Fig 13, it can be seen that there are differences in the strength of the force chain and the positional distribution of the force chain after rock damage under Y-cracks with different deflection angles. Under the same loading conditions, the strong force chains of the intact specimens are mainly distributed in the spike-like damage zone and the lower left corner; the strong force chains of the rock specimens with prefabricated Y-shaped crack are mainly distributed near the Y-shaped crack, which indicates that stress concentration occurs near the Y-shaped crack. The distribution of the weak force chains of the intact specimens is relatively decentralized; the weak force chains of the rock specimens containing prefabricated Y-shaped crack s are mainly distributed on the left and right sides of the specimens, and the weak force chains have less influence on the damage of the specimens. It can be seen that when the force chain is subjected to more than its strength will be fractured, the strong force chain breakage leads to the failure of the force between the particles, as shown in the figure of the destruction of the specimen fissure at the force chain have been fractured.

## Discussion

The research value of this paper is in the field of mining. The influence of Y-shape fracture on rock failure is directly related to the stability and safety of mine. Our research reveals the expansion law and failure mechanism of Y-shaped crack under stress conditions, which provides a basis for mining engineering to formulate more reasonable mining sequence, select more effective support measures and optimize blasting parameters. In civil engineering, the mechanical properties of rocks are the key factors that determine the stability and durability of engineering structures. Our research shows that the existence of Y-shaped cracks can significantly reduce the strength and stiffness of rocks, and affect the bearing capacity and deformation characteristics of engineering structures. Therefore, the influence of Y-shape crack should be fully considered in geotechnical engineering design, and the overall stability of engineering structure should be improved by strengthening foundation treatment, optimizing structural design or adopting special reinforcement measures. For geological risk assessment, it is an important means to prevent geological disasters and protect people’s life and property safety. Our research provides a new perspective and method for geological risk assessment. By analyzing the relationship between Y-shaped cracks and geological disasters (such as landslide, collapse, etc.), the occurrence probability and scale of geological disasters can be more accurately evaluated, and scientific basis for formulating effective disaster prevention and reduction measures can be provided.

When the PFC simulation test results are used for analysis, it is found that there are some deviations from the laboratory test results, such as the direction of crack growth and the failure mode of the specimen. This is because the laboratory test is carried out under actual physical conditions, which can truly reflect the mechanical response of the material in a specific environment. However, laboratory tests are often affected by many factors, such as the accuracy of the test equipment, the standardization of the test operation, and the stability of the test environment. All these factors may have some influence on the test results, resulting in some discreteness and uncertainty in the laboratory test results. In addition, there may be differences between PFC simulation test and laboratory test in boundary conditions, loading methods, sample sizes, etc. This results in some deviation between PFC simulation test and laboratory test.

## Conclusion

In this paper, the mechanical properties of rock damage containing prefabricated Y-shaped crack are investigated using a combination of indoor tests and numerical simulation research methods. The following conclusions are mainly obtained:

1. With the increase of Y-shaped crack Angle, the peak strength of the sample showed a trend of first decreasing, then increasing and then decreasing during the evolution process, and different angles directly affected the mechanical properties of the sample.
2. The crack initiation of the sample is located at the tip of the prefabricated Y-shaped crack, and the newly generated crack is mainly shear, and the crack initiation location and propagation evolution change with the change of the Y-shaped crack Angle.
3. The failure of the sample is mainly caused by shear crack, and cracks at different angles directly affect the generation and evolution of new cracks in the sample.

## Supporting information

S1 Data (XLSX)
